# Supplementary material for: Influence of Acute and Chronic Graft-Versus-Host Disease on Persistence of Antibodies against Measles, Mumps, Rubella and Varicella in the First Year after Autologous or Allogeneic Hematopoietic Stem Cell Transplantation
Source: Vaccines (Basel). 2023 Mar 14;11(3):656. doi: 10.3390/vaccines11030656 (PMC10052354; doi:10.3390/vaccines11030656)
Supplement: Supplementary file 1 [file vaccines-11-00656-s001.zip › vaccines-2236654-supplementary.docx]

**Supplementary Figure S1.** Time course of absolute lymphocyte counts at all seven time points for allogeneic (squares) and autologous (circles) patients. Time points: on admission (7 days before HSCT; T-1), on the day of HSCT (T0), during aplasia (absolute neutrophil count <0.5 G/L; T+1), at engraftment (T+2, neutrophil count >0.5G/l), 1 month after HSCT (T+3), 3 to 6 months after HSCT (T+4), and 6 to 12 months after HSCT (T+5).


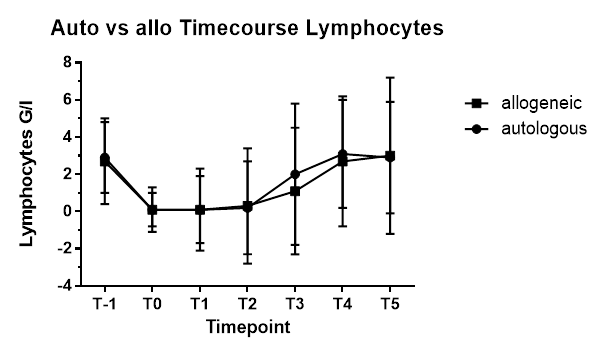


**Supplementary Figure S2.** Comparison of antibody titers at baseline (T -1) and at time of highest severity of acute GvHD (T2 or T3) in allogeneic patients with acute GvHD.

**Supplementary Figure S3.** Comparison of antibody titers at baseline (T -1) and at time of highest severity of chronic GvHD (T4 or T5) in allogeneic patients with chronic GvHD.
